# Four Tomato FLOWERING LOCUS T-Like Proteins Act Antagonistically to Regulate Floral Initiation

**DOI:** 10.3389/fpls.2015.01213

**Published:** 2016-01-11

**Authors:** Kai Cao, Lirong Cui, Xiaoting Zhou, Lin Ye, Zhirong Zou, Shulin Deng

**Affiliations:** ^1^State Key Laboratory of Crop Stress Biology for Arid Areas, Horticulture College, Northwest A&F UniversityYangling, China; ^2^Laboratory of Plant Molecular Biology, Rockefeller UniversityNew York, NY, USA

**Keywords:** tomato, floral repressor, floral activator, PEBP protein, FT-like genes, phytochromes

## Abstract

The transition from vegetative growth to floral meristems in higher plants is regulated through the integration of internal cues and environmental signals. We were interested to examine the molecular mechanism of flowering in the day-neutral plant tomato (*Solanum lycopersicum* L.) and the effect of environmental conditions on tomato flowering. Analysis of the tomato genome uncovered 13 PEBP (phosphatidylethanolamine-binding protein) genes, and found six of them were *FT*-like genes which named as *SlSP3D, SlSP6A, SlSP5G, SlSP5G1, SlSP5G2*, and *SlSP5G3*. Six FT-like genes were analyzed to clarify their functional roles in flowering using transgenic and expression analyses. We found that SlSP5G, SlSP5G2, and SlSP5G3 proteins were floral inhibitors whereas only SlSP3D/SFT (*SINGLE FLOWER TRUSS*) was a floral inducer. *SlSP5G* was expressed at higher levels in long day (LD) conditions compared to short day (SD) conditions while *SlSP5G2* and *SlSP5G3* showed the opposite expression patterns. The silencing of *SlSP5G* by VIGS (Virus induced gene silencing) resulted in tomato plants that flowered early under LD conditions and the silencing of *SlSP5G2* and *SlSP5G3* led to early flowering under SD conditions. The higher expression levels of *SlSP5G* under LD conditions were not seen in *phyB1* mutants, and the expression levels of *SlSP5G2* and *SlSP5G3* were increased in *phyB1* mutants under both SD and LD conditions compared to wild type plants. These data suggest that *SlSP5G, SlSP5G2*, and *SlSP5G3* are controlled by photoperiod, and the different expression patterns of *FT*-like genes under different photoperiod may contribute to tomato being a day neutral plant. In addition, PHYB1 mediate the expression of *SlSP5G, SlSP5G2*, and *SlSP5G3* to regulate flowering in tomato.

## Introduction

In flowering plant, the timing of the transition from vegetative to reproductive phase is a major event in the plant life cycle. Both physiological and genetic studies have revealed the complexity of mechanisms that tightly control switch from vegetative to reproductive growth in the apical meristem (Bernier et al., 1993; Shalit et al., 2009). The phosphatidylethanolamine-binding proteins (PEBPs), found in both angiosperms and gymnosperms, have evolved to become both activators and repressors of flowering and they can be classified into three clades (Gyllenstrand et al., 2007; Karlgren et al., 2011). An example of this functional diversification is seen in the six PEBP family members of Arabidopsis. FLOWERING LOCUS T (FT) and TWIN SISTER OF FT (TSF), which belong to the FT-like clade, function as flowering activators, TERMINAL FLOWER 1 (TFL1), BROTHER OF FT AND TFL1 (BFT), and ARABIDOPSIS THALIANA CENTRORADIALIS (ATC), which classify to the TFL1-like clade, are usually flowering repressors, and MOTHER OF FT AND TFL1(MFT), which defines the MFT-like clade, is predominantly a floral promoter (Karlgren et al., 2011).

In Arabidopsis, a long-day plant, FT is expressed in leaf phloem companion cells. This protein which triggers floral development in the shoot apical meristem (SAM) under long day (LD) conditions is a major output of the photoperiod pathway and controls floral transition in response to the changes in day length (Kardailsky et al., 1999; Kobayashi et al., 1999). CONSTANS (CO) encodes a zinc finger protein and promotes flowering under LD conditions (Putterill et al., 1995). In LD conditions, *FT* is activated by CO (Samach et al., 2000), and the FT protein then interacts with a novel endoplasmic reticulum membrane protein called FT-INTERACTING PROTEIN 1 (FTIP1; Liu et al., 2012). Following the interaction FT is transported from the companion cells to the sieve elements and entered the SAM by mass flow, where it associates with the basic leucine zipper domain (bZIP) transcription factor FD to activate downstream targets such as SUPPRESSOR OF OVEREXPRESSION OF CONSTANS 1 (SOC1) and the floral meristem identity gene APETALA 1 (AP1; Abe et al., 2005; Wigge et al., 2005; Corbesier et al., 2007). Also a PEBP family protein TSF probably acts in a similar way to FT (Yamaguchi et al., 2005). In SD conditions, Arabidopsis flowering is controlled by a gibberellin pathway, which promotes flowering through the activation of the flower meristem identity gene LEAFY (LFY) with no involvement of any PEBP family proteins (Moon et al., 2003). In SD plant rice, Hd3a, a FT homolog promotes flowering under SD conditions (Komiya et al., 2008, 2009). In the day-neutral plant tomato, the homolog of FT, SlSP3D/SFT (SINGLE-FLOWER TRSS), has been shown to encode the mobile florigen signal and promote tomato flowering (Molinero-Rosales et al., 2004).

Although almost all FT-like proteins act as floral activators an antagonistically functional switch has occurred in *Beta vulgaris* (sugar beet) and *Nicotiana tabacum* (tobacco) because of gene duplication event(s) generating other paralog(s). In sugar beet, BvFT1 protein acts as an inhibitor in floral development whereas another FT-like protein BvFT2 works as a promoter (Pin et al., 2010). Substitutions of specific amino acids can convert BvFT1 to a floral inducer and BvFT2 into a floral repressor (Pin et al., 2010). In tobacco, four *FT*-like proteins, NtFT1, NtFT2, and NtFT3 proteins are floral inhibitors whereas only NtFT4 is a floral inducer (Harig et al., 2012). These data suggest that some FT-like proteins, which are evolutionarily more related to FT than to TFL1/CEN, have evolved into flowering repressors.

Phytochromes are primary photosensory receptors that perceive red and far-red light of higher plants. These photochromic proteins exist in two photo-interconvertible isomeric forms: the red light absorbing form and the far-red light absorbing form (Hughes and Lamparter, 1999). Arabidopsis has five phytochrome genes, *PHYA* to *PHYE*, which encode the apoproteins of PHYA to PHYE, respectively (Quail et al., 1995). *PHYB* plays an inhibitory role in floral initiation in Arabidopsis; the *phyB* mutant flowered earlier than WT in both LD and SD conditions, but the early-flowering phenotype of the *phyB* mutant is more pronounced in SD than in LD conditions (Goto et al., 1991; Mockler et al., 1999). *phyB* mutations of the LD pea plant (Weller and Reid, 1993), SD plant sorghum (Childs et al., 1997), and rice (Izawa et al., 2002) showed early-flowering and decreased photoperiodic sensitivities. *PHYB* delays flowering by suppressing the expression of *FT* in Arabidopsis (Endo et al., 2005) and *Hd3a* in rice (Izawa et al., 2002). Tomato contains five phytochrome genes, named *PHYA, PHYB1, PHYB2, PHYE*, and *PHYF* (Hauser et al., 1997). The tomato *PHYB1* is mainly involved in the de-etiolation response of seedlings, unfolding of the hypocotyl hook, cotyledon expansion, hypocotyl elongation, and anthocyanin accumulation (Kerckhoffs et al., 1997; Weller et al., 2000). However, the function of phytochromes in tomato flowering have not yet been reported.

Tomato is a photoperiod-insensitive, perennial in its native habit. The flowering time of tomato is measured by the number of leaves in the initial segment, which is rather stable under various environmental conditions (Kinet, 1977). Here, we performed expression and transgenic studies to clarify the functional roles of four expressed *FT*-like genes in tomato. One of the *FT*-like genes has already been identified by Molinero-Rosales et al. (2004), whereas the other three genes have not been studied. Here, we demonstrate the functional differentiation between these genes in controlling flowering through overexpression in Arabidopsis and VIGS-mediated knocking down in tomato. Our data suggest that among four expressed *FT*-like proteins, three of them act as floral repressors and only one of them function as a floral promoter. We also showed the expression profiles of tomato *FT*-like genes under LD and SD conditions in tomato wild-type (WT) and *phy* mutants. The evolution of antagonistic *FT*-like paralogs may be a common strategy in *Solanaceous* plants to fine-tune floral development in response to internal and environmental cues.

## Materials and methods

### Plant material and growth conditions

We used cv. MoneyMaker (*Solanum lycopersicum* L.) wild type (WT) as control in this study, and *phyA, phyB1, phyB2*, and *phyB1B2* mutants in the MoneyMaker background were provided by the Tomato Genetic Resource Center (Department of Vegetable Crops, University of California, Davis) and their TGR accession numbers were LA4356, LA4357, LA4358, and LA4364, respectively. Tomato seeds were soaked in 50% bleach for 30 min. After the treatment, seeds were rinsed thoroughly in running water, then sown directly on a germination paper and incubated at 25°C. After germination, seedings were sowed onto commercial substrate and grown in a growth chamber under LD (16 h of light/8 h of dark) conditions or SD (8 h of light/16 h of dark) at 300 μmol m^−2^ s^−1^ and 25°C (both day and night).

To study the spatial expression patterns of *FT*-like genes, we extracted total RNA from leaf, apex, stem, flower, and root tissues, pooled from three 7-week-old plants. For diurnal changes in the expression of *FT*-like genes, leaves were harvested every 4 h for 24 h (0, 4, 8, 12, 16, 20, and 24 h), pooled from 3 third leaves of 5-week old plants. To study the effect of photoperiod on the expression of these genes, 5-week old uniform plantlets were transferred from LD conditions to SD conditions and reversely. Three different leaves at the same level were harvested 1, 2, and 3 day after the transfer.

### Phylogenetic analysis

Tomato protein sequences of the PEBP family members were downloaded from https://solgenomics.net/, *Arabidopsis thaliana* PEBP family members were downloaded from https://www.arabidopsis.org/, tobacco FT-like proteins reported by Harig et al. (2012) were download from https://solgenomics.net/, and sugar beet FT-like proteins reported by Pin et al. (2010) were download from http://www.ncbi.nlm.nih.gov/. Protein sequences were aligned using the maximum-likelihood method implemented in ClustalW software (Thompson et al., 1994). An N-J tree was produced from the results of 1000 bootstrap replicates using the ClustalW program.

### Gene expression studies

Total RNA was extracted using an RNeasy Plant Mini Kit (Qiagen) following the manufacturer's instructions. cDNA synthesis was performed by using the SuperscriptIII First strand synthesis system (Invitrogen) following the manufacturer's instructions. Real-time PCR was performed using SYBR Premix Ex Taq (TAKARA) in a Biorad CFX96 realtime PCR system. *ACTIN* was used as an internal control. The primers used were listed in Supplementary Table S1. Real-time quantitative PCR was repeated with three biological replicates, and each sample was assayed in triplicate by PCR.

### Plasmid constructs and plant transformation

The ORFs of *SlSP3D*(*Solyc03g063100*), *SlSP5G*(*Solyc05g053850*), *SlSP5G2*(*Solyc11g008640*), *SlSP5G3*(*Solyc11g008650*) were amplified by PCR, cloned in *pENTR/3C* vector (Invitrogen) and then transferred into *pBCO-DC* by recombination (Jang et al., 2007) using LR Clonase enzyme (Invitrogen). The resultant plasmid was used to transform *A. thaliana* (Col-0) plants by the *Agrobacterium tumefaciens* strain GV3101-mediated floral dip method (cite the original ref as well; Zhang et al., 2006). Transformed plants were selected on 0.8% agar media containing Murashige and Skoog salts, 0.5 g/L MES, and 10 g/L Sucrose and containing 10 μg/L basta. Arabidopsis plants were grown in a growth chamber under LD conditions at a light intensity of 100 μmol m^−2^ s^−1^ at 20°C (day and night).

### Virus-induced gene silencing (VIGS) in tomato plants

*pTRV1* (*pYL192*) and *pTRV2* (*pYL156*) vectors had been described in Liu et al. (2002). The *pYL170 TRV2* vector was derived by cloning a *PstI*-blunt-*DraIII* fragment of *pYL156* into *EcoRI*-blunt-*DraIII*-cut *pCAMBIA3301*. This vector was identical to *pYL156*, except for a plant selection marker. To generate *pTRV2-SlPDS, pTRV2-SlSP5G, pTRV2SlSP5G2*, and *pTRV2SlSP5G3*, a cDNA fragment was PCR amplified using a tomato ecotype MoneyMaker cDNA library and primers were described in Supplementary Table S1. The resulting PCR products were cloned into *EcoRI*-*BamH*I-cut *pTRV2* (*PYL170*).

One-week-old tomato seedlings were used for the VIGS assay, *pTRV1* and *pTRV2* or its derivatives were introduced into *A. tumefaciens* strain GV3101 and the Agrobacterial strains mixed. A 5-mL culture was grown for 16 h at 28°C in 50 mg/L gentamycin and 50 mg/L kanamycin. The next day, the culture was inoculated into 30 mL of Luria-Bertani medium containing antibiotics, 10 mM MES, and 20 mM acetosyringone. The culture was grown 16 h in a 28°C shaker (200 r.p.m). *A. tumefaciens* cells were harvested and resuspended in infiltration media (10 mM MgCl_2_, 10 mM MES, and 200 mM acetosyringone), adjusted to an OD600 of 1.5, and left at room temperature for 3–4 h. Agroinfiltration was performed with a needleless 1-mL syringe into two tomato cotyledons (Velásquez et al., 2009).

## Results

### Identification and phylogenetic classification of tomato FT-like genes

**Figure 1:**
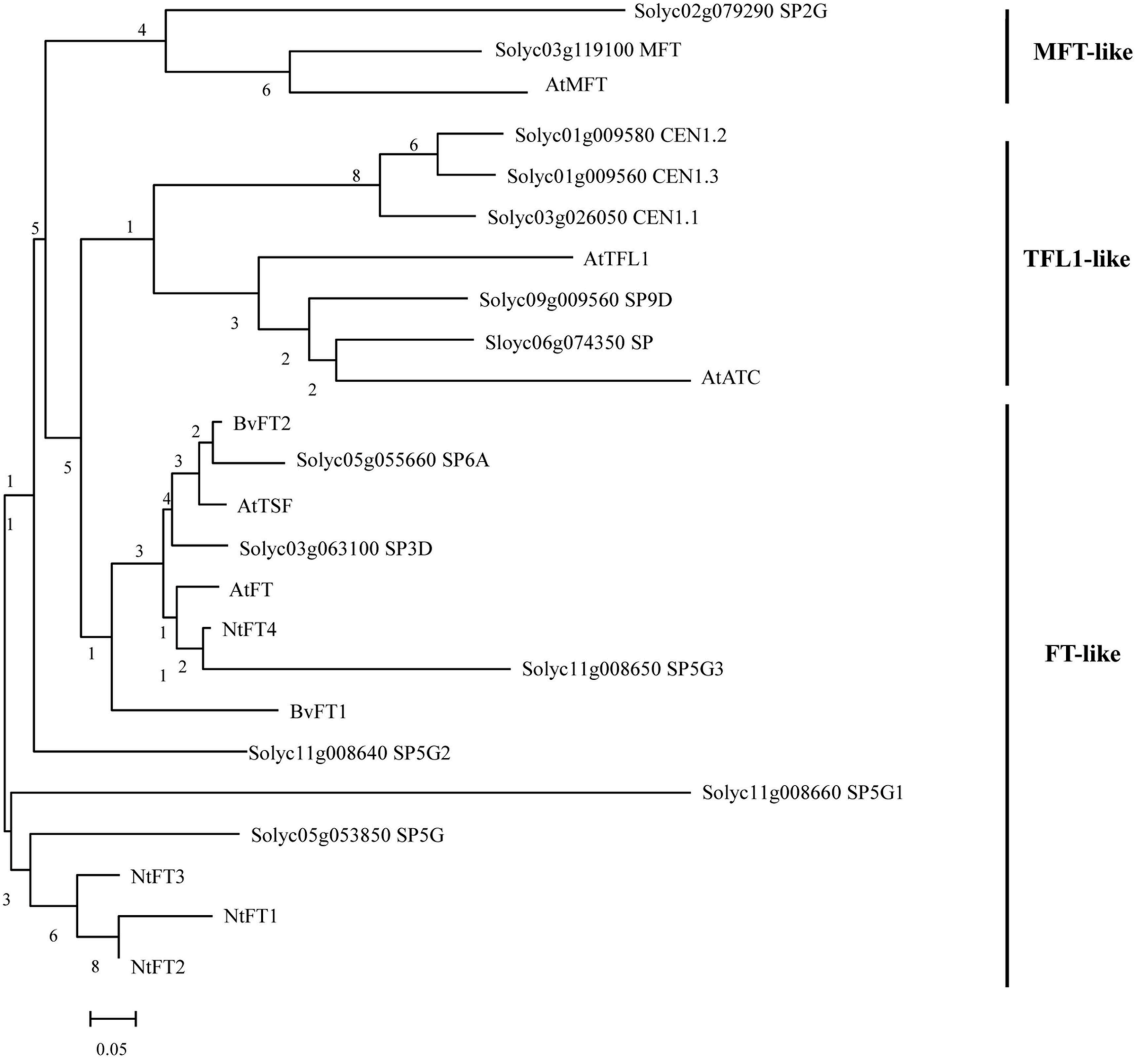
**Phylogenetic tree of PEBP proteins from tomato, sugar beet, tobacco, and Arabidopsis**. Sequences were aligned with ClustalX and the results are displayed graphically using TreeView. The tomato PEBP genes are grouped into three major clades: the FT-like, TFL-like, and MFT-like clades. AtFT, *Arabidopsis thaliana* FLOWERING LOCUS T; AtTSF, *A. thaliana* TWIN SISTER OF FT; AtTFL1, *A. thaliana* TERMINAL FLOWER 1; AtATC, *A. thaliana* ARABIDOPSIS THALIANA CENTRORADIALIS; AtMFT, *A. thaliana* MOTHER OF FT AND TFL1; BvFT1-2, *Beta vulgaris* FLOWERING LOCUS T 1-2; NtFT1-4, *Nicotiana tabacum* FLOWERING LOCUS T 1-4.

To identify FT-like proteins encoded by the tomato genome, the amino acid sequence of Arabidopsis FT protein was used to perform a BLAST survey against the tomato whole-genome database (https://solgenomics.net/). A total of 13 predicted PEBP genes were identified and annotated. In a previous study, the plant PEBP family could be classified into three main clades, described as FT-like, TFL1-like, and MFT-like (Chardon and Damerval, 2005). To evaluate the evolutionary relationships among the tomato, tobacco, sugar beet, and Arabidopsis FT-like proteins, specific and combined phylogenetic analysis based on their amino acid sequence were performed. We created a maximum-likelihood tree from an alignment of the 13 tomato PEBP proteins, the Arabidopsis 6 PEBP proteins, the tobacco FT-like (NtFT1-NtFT4) proteins and the sugar beet BvFT1 and BvFT2 proteins. Figure 1 shows that there were six *FT*-like genes, five *TFL1*-like genes, and two *MFT*-like genes in the tomato PEBP family. PEBP family proteins contained two key motifs which are a putative ligand-binding pocket and an external loop. Protein sequence alignment also revealed a change of an amino acid residue from Tyr in tomato FT-like proteins to His in TFL1-like at the entrance of the binding pocket (Supplementary Figure S1), This amino acid residue in part determines the functional difference between FT and TFL1 in Arabidopsis (Hanzawa et al., 2005). Another amino acid residue was changed from Gln in tomato FT-like proteins to Asp in TFL1-like at the external loop encoded by the fourth exon (Supplementary Figure S1). This was another critical residue for the functional difference between FT and TFL1 in Arabidopsis (Ahn et al., 2006). These results suggest that *SlSP3D, SlSP6A, SlSP5G, SlSP5G1, SlSP5G2*, and *SlSP5G3* are *FT*-like genes.

Nucleotide sequence comparisons between genomic and predicted CDS allowed the identification of the exon-intron structures of tomato PEBP genes. Tomato PEBP genes showed conserved genomic organization and the exons were placed in identical positions relative to the amino acid sequence of the Arabidopsis PEBP genes family, except for *SlSP5G1* and *SlSP5G3* (Supplementary Figure S2). The length of exons was quite conserved compared among tomato FT-related genes themselves and with *Arabidopsis* FT-related genes, but the introns differed in length. For the FT-like genes exon-intron structures, *SlSP6A* and *SlSP5G1* were truncated by a premature stop codon in their last exon and there was only one 222 exon without intron for *SlSP5G3*. In sugar beet and tobacco, FT-like protein could be further divided into floral promoters and floral repressors.

### Expression pattern of FT-like genes in different organs under LD and SD conditions

**Figure 2:**
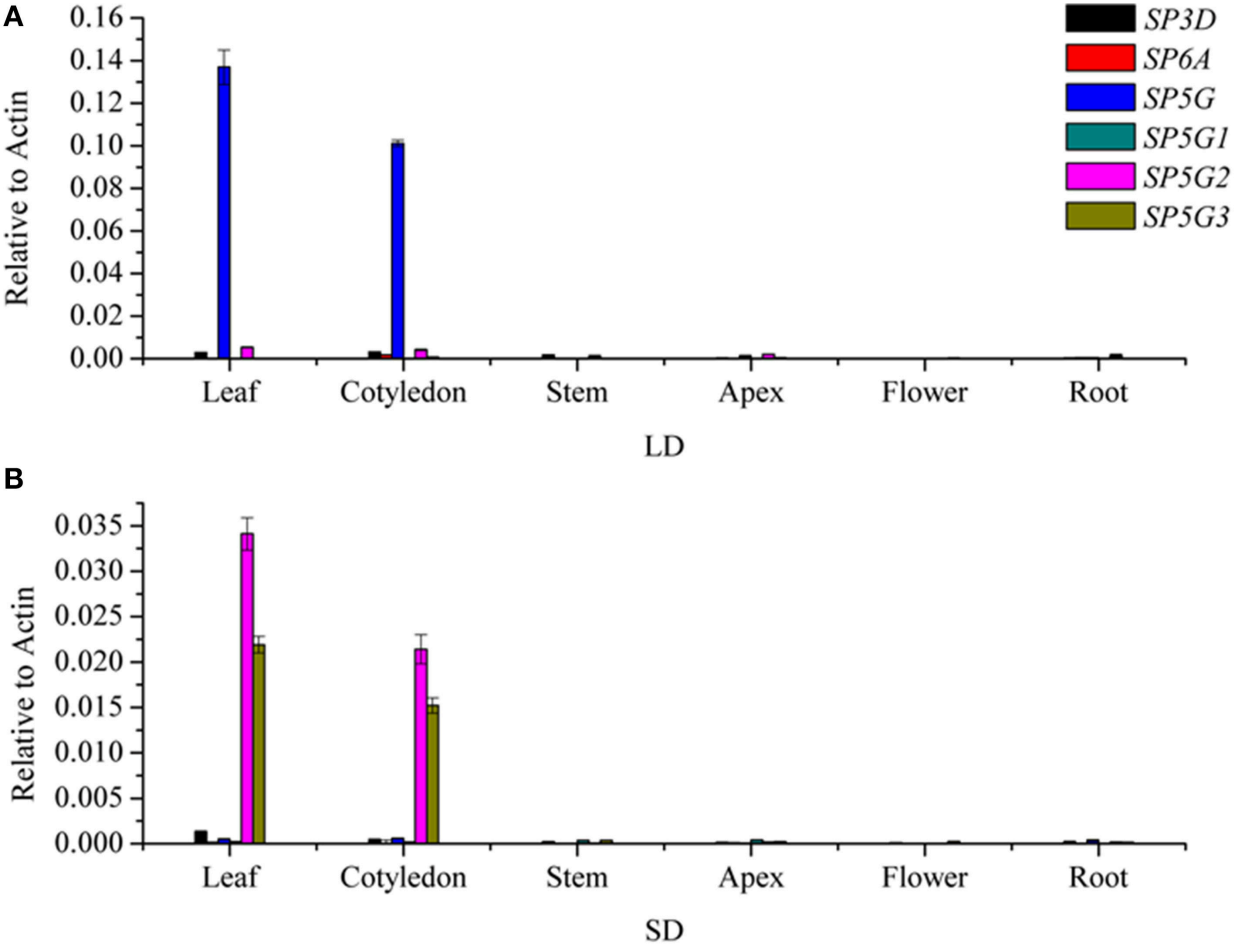
**Transcription analyses of tomato *FT*-like genes in different organs**. *FT*-like genes expression analyzed by qRT-PCR using RNA extracted from leaf, cotyledon, stem, apex, flower, and root in 7-week old tomato plants grown under LD condition **(A)** and SD condition **(B)**. Expression of the tomato *ACTIN* gene was used as a reference. All data are showed as mean ±SE of three independent pools of extracts. Three technical replicates were performed for each extract.

To investigate the roles of the six tomato *FT*-like genes in flowering, we first monitored their expression levels in different organs. We isolated total RNA from the leaf, cotyledon, apex, stem, flower, and root tissues of 7-week-old tomato plants growth under LD and SD conditions. We compared the expression levels of *SlSP3D, SlSP6A, SlSP5G, SlSP5G1, SlSP5G2*, and *SlSP5G3* with those of the housekeeping gene *ACTIN* by qRT-PCR. No expression was detected for *SlSP6A* and *SlSP5G1* in all tissues. Considering there are premature stop codons in their last exons (Carmel-Goren et al., 2003; Consortium, 2012), these two genes probably do not encode functional proteins and are in fact pseudogenes. *SlSP3D, SlSP5G, SlSP5G2*, and *SlSP5G3* are mainly expressed in leaf and cotyledon under both LD and SD conditions (Figures 2A,B). A much higher expression level of *SlSP5G* was observed under LD conditions compared to SD conditions and *SlSP5G2* and *SlSP5G3* displayed an opposite expression pattern (Figures 2A,B). Under SD conditions, the number of leaves on the tomato main stem at flowering was eight on average, while this number increased to nine under LD conditions (**Figure 5A**).

### Diurnal rhythmic expression patterns of FT-like genes

**Figure 3:**
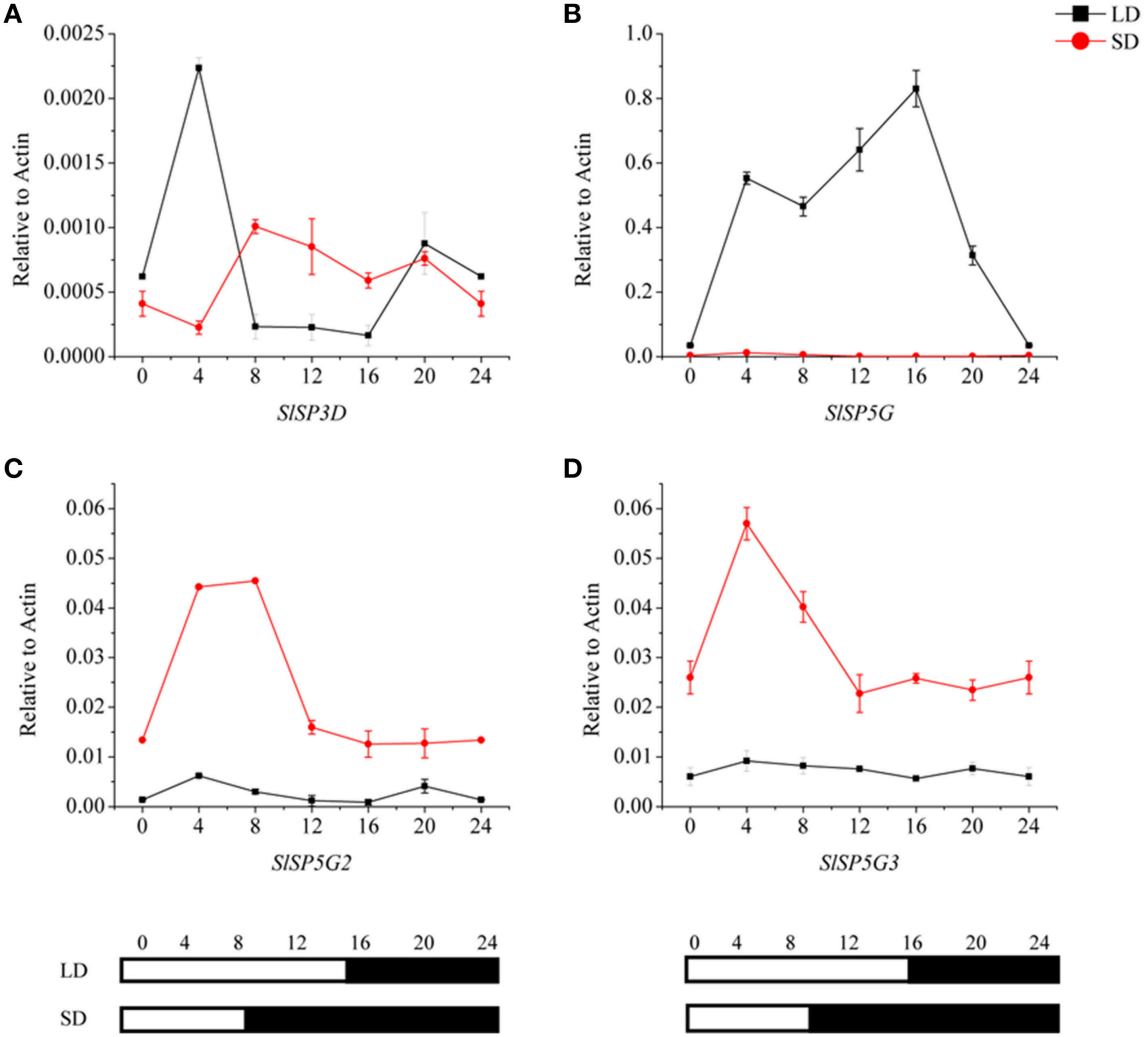
**Diurnal expression patterns of tomato *FT*-like genes, *SlSP3D* (A), *SlSP5G* (B), *SlSP5G2* (C), and *SlSP5G3* (D) under LD and SD conditions**. The black line represents LD condition and the red line represents SD condition. Leaves were harvested from plants at 4-h intervals throughout a light cycle. The vertical axis shows relative mRNA levels of *FT*-like genes to *ACTIN* expression levels. All data are showed as mean ±SE of three independent pools of extracts. Three technical replicates were performed for each extract. White and black bars at the bottom indicate light and dark periods, respectively.

To investigate the relationships among the four expressed FT-like genes, *SlSP3D, SlSP5G, SlSP5G2*, and *SlSP5G3*, we examined their diurnal expression patterns using the third leaves of 5-week-old seedlings. We performed qRT-PCR analyses using RNA from tomato plants grown in a LD diurnal cycle or a SD diurnal cycle. The expression of *SlSP3D* peaked at 4 h after dawn under LD conditions (Figure 3A) confirming previous results (Shalit et al., 2009). Our results also revealed that *SlSP5G* was transcribed at dawn and its expression peaked at the end of the day under LD conditions (Figure 3B). Under SD conditions, *SlSP5G* was constantly expressed at a lower level compared with its expression under LD conditions (Figure 3B). The expression pattern of *SlSP5G2* was different from that of *SlSP5G*, which showed a higher expression level under SD conditions, with expression peaking after 4 h of light under SD conditions (Figure 3C). On the other hand, *SlSP5G3* showed nearly the same diurnal oscillation pattern as *SlSP5G2*, and it peaked at 4 h after light under SD conditions (Figure 3D).

### Tomato FT-like genes have antagonistic functions in floral development in transgenic arabidopsis plants

According to previous studies, *SlSP6A* and *SlSP5G1* were not expressed in tomato plants (Abelenda et al., 2014; Figures 2A,B) and consistent with this result we failed to clone *SlSP6A* and *SlSP5G1* from our tomato cDNA library. To investigate the functions of other *FT*-like genes in tomato flowering, *SlSP3D, SlSP5G, SlSP5G2*, and *SlSP5G3* were transferred into Arabidopsis plants under the control of a cauliflower mosaic virus (CaMV) *35S* promoter.

**Figure 4:**
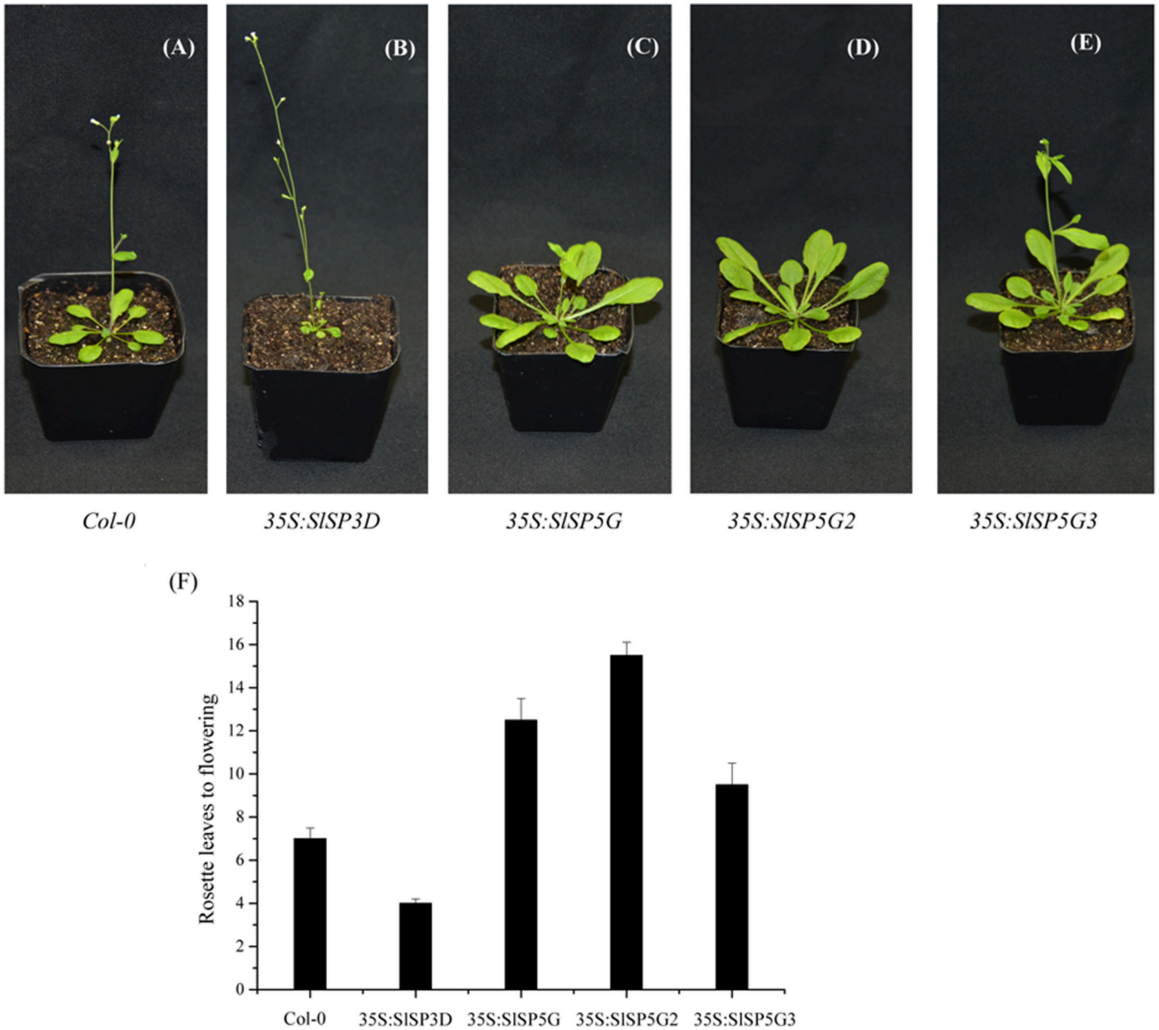
**Overexpression of *SlSP3D* in transgenic Arabidopsis promotes flowering, but overexpression of *SlSP5G*, *SlSP5G2*, and *SlSP5G3* in transgenic Arabidopsis causes a delay in flowering**. **(A–E)** Wild type Col-0 **(A)** or representative transgenic Arabidopsis line overexpressing *SlSP3D* **(B)**, *SlSP5G* **(C)**, *SlSP5G2* **(D)**, and *SlSP5G3* **(E)** were grown under LD conditions. **(F)** The rosette leaves produced from SAM prior to flowering under LD conditions. The rosette leaves data are showed as mean ±SE of eight plants in each overexpression line. qRT-PCR data are showed as mean ±SE of three independent pools of extracts. Three technical replicates were performed for each extract.

Overexpressing *SlSP3D* led to early flowering in transgenic Arabidopsis (Figure 4B) cofirming previous report that *SlSP3D* was a flowering promoter (Molinero-Rosales et al., 2004). Overexpression of *SlSP5G, SlSP5G2*, and *SlSP5G3* delayed flowering in transgenic Arabidopsis plants compared to wild-type controls (Figures 4A,C–E). The number of rosette leaves before flowering was seven in Col-0 under LD conditions. However, this number decreased to four in *SlSP3D* overexpressing plants (line 1), increased to 9.5 in *SlSP5G3* overexpressing plants (line 1), 12.5 in *SlSP5G* overexpressing plants (line 2) and 15.5 in *SlSP5G2* (line 2) overexpressing plants under LD conditions (Figure 4F). There were four overexpressing *SlSP3D, SlSP5G, SlSP5G2*, and *SlSP5G3* lines, respectively, and the number of rosette leaves before flowering in the other overexpressing *SlSP3D, SlSP5G, SlSP5G2*, and *SlSP5G3* lines were shown in Supplementary Figure S3. These results indicate that *SlSP3D* is a floral promoter, and *SlSP5G, SlSP5G2*, and *SlSP5G3* are floral repressors.

### The effect of photoperiod on the expression of SLSP3D, SLSP5G, SLSP5G2, and SLSP5G3 genes

**Figure 5:**
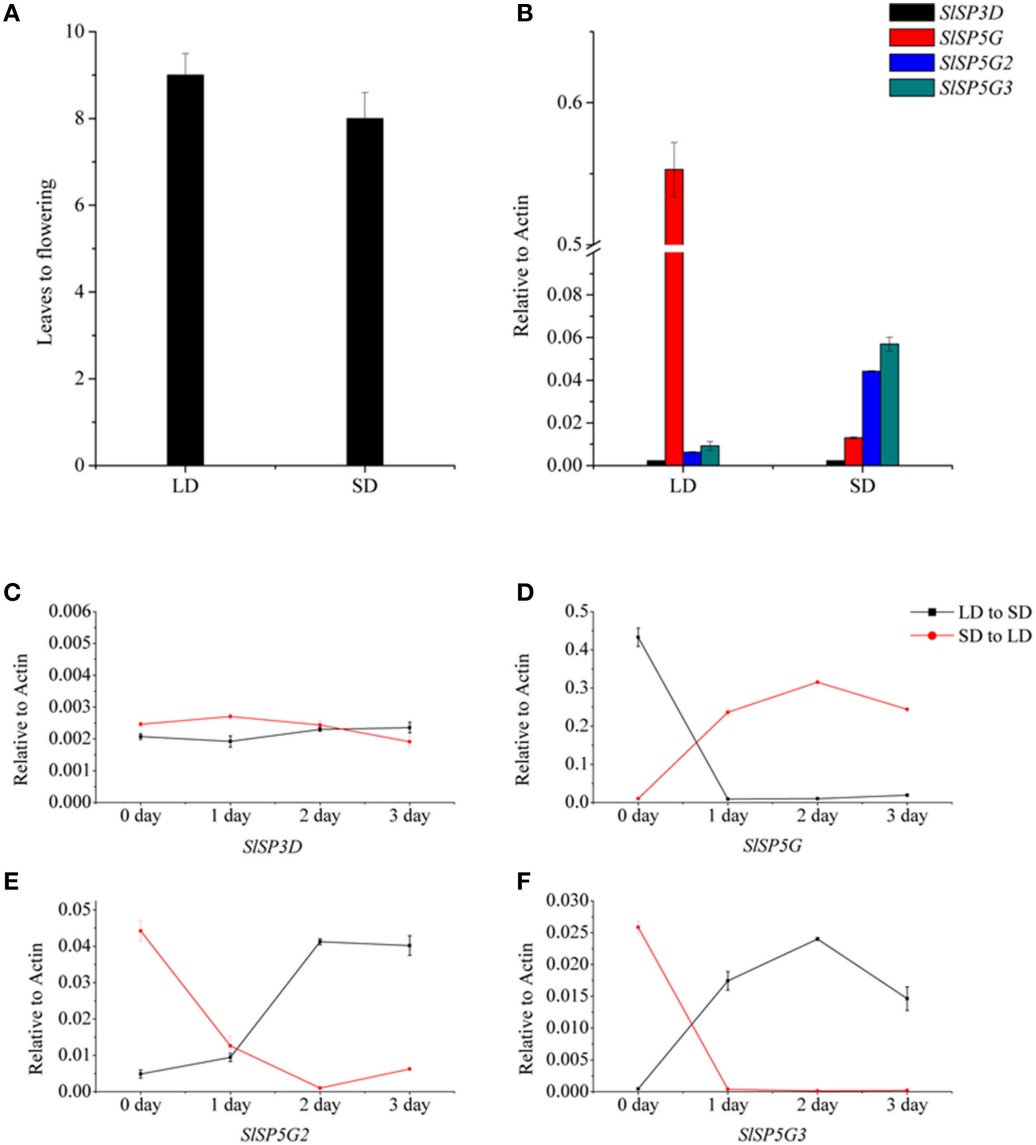
**The number of leaves at flowering of tomato plants under different photoperiod and transcription analysis of tomato *FT*-like genes by qRT-PCR in plants exposed to LD or SD conditions**. **(A)** The number of leaves at flowering of tomato plants under LD or SD conditions. Data showed are mean ±SE (*n* = 10 plants). **(B)** The expression of *SlSP3D, SlSP5G, SlSP5G2*, and *SlSP5G3* under LD and SD conditions. **(C–F)** Expression patterns of *SlSP3D* **(C)**, *SlSP5G* **(D)**, *SlSP5G2* **(E)**, and *SlSP5G3* **(F)** of tomato plants transferred from SD to LD condition, and vise-versa. All data are expressed as means ±SE of three independent pools of extracts. Three technical replicates were performed for each extract.

Figure 5B shows that *SlSP5G* expression increased under LD conditions, while *SlSP5G2* and *SlSP5G3* expression increased under SD conditions. These results suggested that *SlSP5G, SlSP5G2*, and *SlSP5G3* were targets of photoperiodic regulation. Therefore, we determined the expression levels of *SlSP3D, SlSP5G, SlSP5G2*, and *SlSP5G3* in tomato plants grown under LD conditions for 4 weeks and then transferred to SD conditions for 3 days, and vise-versa. There was no change of *SlSP3D* expression when tomato plants were transferred from LD conditions to SD conditions or from SD conditions to LD conditions (Figure 5C). Downregulation of *SlSP5G* and upregulation of *SlSP5G2* and *SlSP5G3* were apparent after tomato plants were transferred from LD conditions to SD conditions (Figures 5D–F). With tomato plants transferred from SD conditions to LD conditions, we found a directly increase of *SlSP5G* expression and a decrease of *SlSP5G2* and *SlSP5G3* expression after only one LD photoperiod (Figures 5D–F). These results indicated that *SlSP5G, SlSP5G2*, and *SlSP5G3* are directly regulated by day length.

### Silencing of the tomato SLSP5G, SLSP5G2, and SLSP5G3 genes using TRV-VIGS vector

**Figure 6:**
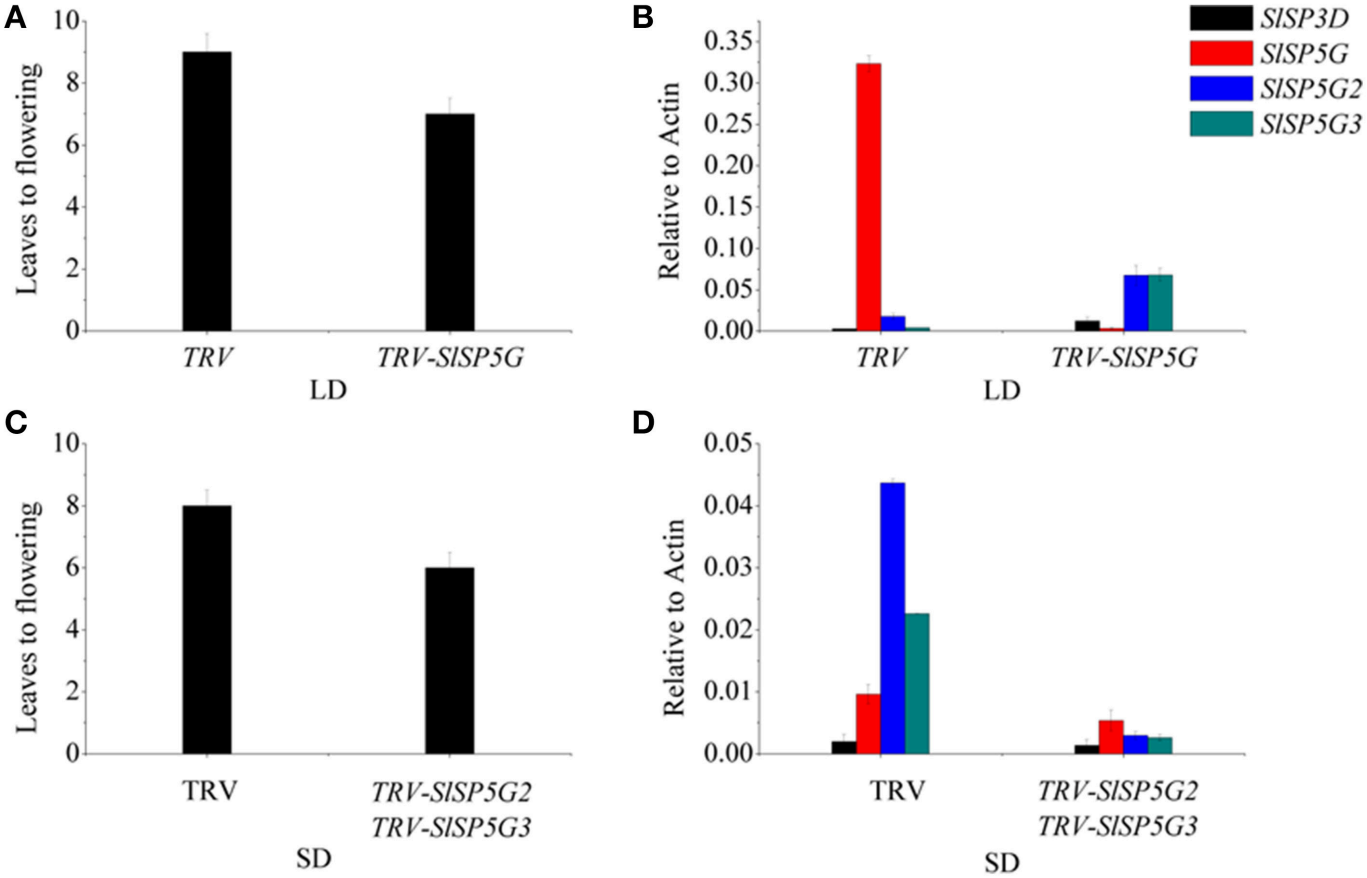
**Flowering time and transcript analysis of VIGS of Sl*SP5G* or Sl*SP5G2* and *SlSP5G3* tomato plants**. **(A)** The number of leaves at flowering of tomato plants when infected by *TRV-SlSP5G* vectors under LD conditions. Data are mean ±SE (*n* = 10 plants). **(B)** The expression of *SlSP3D, SlSP5G, SlSP5G2*, and *SlSP5G3* of tomato plants infected by *TRV-SP5G* vectors under LD conditions. **(C)** The number of leaves at flowering of tomato plants when infected by *TRV-SlSP5G2* and *SlSP5G3* vectors under SD conditions. **(D)** The expression of *SlSP3D, SlSP5G, SlSP5G2*, and *SlSP5G3* of tomato plants infected by *TRV-SP5G2* and *SlSP5G3* vectors under SD conditions. All data are expressed as means ±SE of three independent pools of extracts. Three technical replicates were performed for each extract.

To study the function of *SlSP5G, SlSP5G2*, and *SlSP5G3* in tomato flowering under LD and SD conditions, we constructed a TRV-VIGS vector to suppress the expression of the endogenous *SlSP5G, SlSP5G2*, and *SlSP5G3*. A mixture of Agrobacterium cultures containing *pTRV1* and *pTRV2*, carrying tomato *SlSP5G* (*pTRV2-SlSP5G*), *SlSP5G2* (*pTRV2-SlSP5G2*), or *SlSP5G3* (*pTRV2-SlSP5G3*), were infiltrated into the cotyledon of 1-week-old tomato plants. We also used TRV-VIGS vector to suppress the expression of the endogenous phytoene desaturase gene *(PDS*) as a control. Tomato plants infected with *pTRV-SlPDS* developed a photo-bleached phenotype in the upper leaves 10 days post-agro-infiltration (Supplementary Figure S4). Under LD conditions, the number of leaves on tomato main stem upon flowering was nine on average. However, this number was reduced to seven when the tomato plants were infected with *pTRV1/pTRV2-SlSP5G* (Figure 6A). Sixteen out of twenty tomato plants showed early flowering after infiltration with *pTRV1/pTRV2-SlSP5G* compared with tomato plants infiltrated with *pTRV1/pTRV2*. We also extracted RNA from leaves of early flowering tomato plants to confirm that *SlSP5G* was indeed silenced by qRT-PCR. The primers that anneal to the *SlSP5G* gene outside the region targeted for silencing were used. In early flowering tomato plants infiltrated with *pTRV2-SlSP5G, SlSP5G* expression was reduced significantly compared with the *TRV* infected controls (Figure 6B). The results suggest that SlSP5G is a flowering repressor under LD conditions.

Under SD conditions, the number of leaves on tomato main stem at flowering was eight on average; however, this number was reduced to 6.5 in tomato plants infected with *pTRV1*/*pTRV2-SlSP5G2* and *pTRV1*/*pTRV2-SLSP5G3* (Figure 6C). Fourteen out of twenty tomato plants showed slight early flowering after infiltration with *pTRV1*/*pTRV2-SlSP5G2* and *pTRV1*/*pTRV2-SLSP5G3*. RT-PCR also confirmed the decreased expression of *SlSP5G2* and *SlSP5G3* in infiltrated tomato plants (Figure 6D). These data suggest that *SlSP5G2* and *SlSP5G3* are factors that control tomato flowering under SD conditions.

### Effects of phytochrome B1 on the expression of SLSP3D, SLSP5G, SLSP5G2, and SLSP5G3 genes

**Figure 7:**
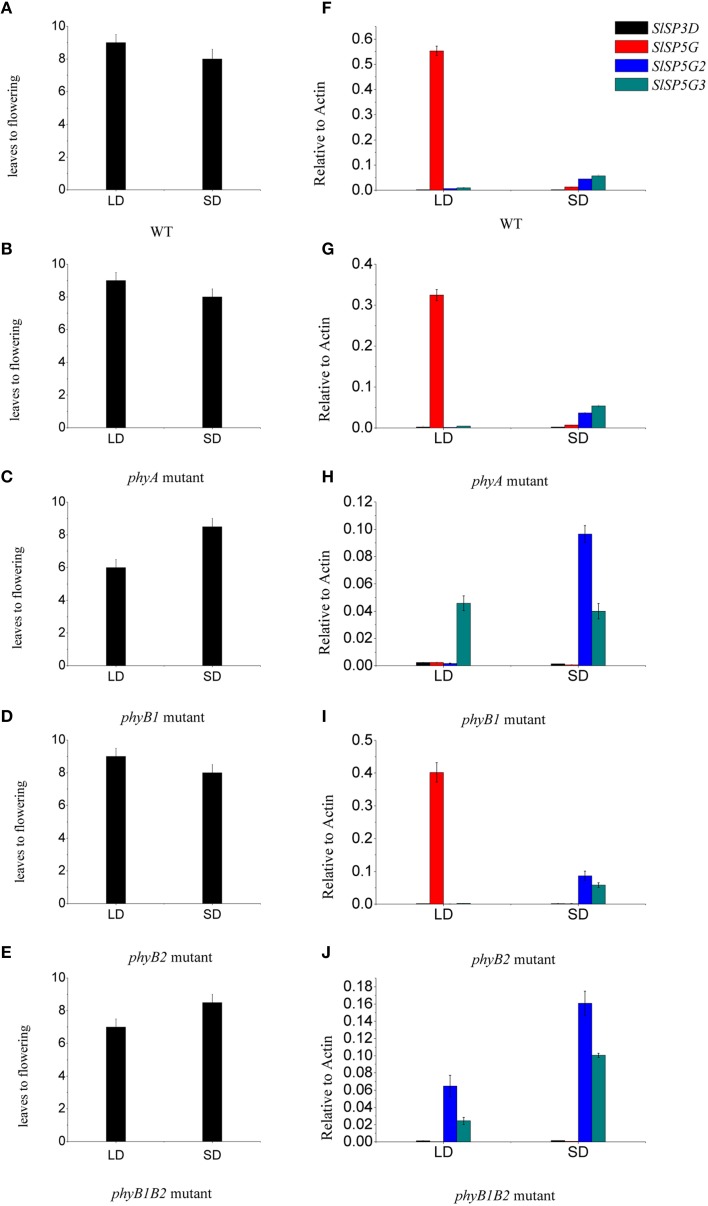
**Phytochrome B1 is responsible for the expression of *SlSP5G* and influences the flowering of tomato plants**. **(A–E)** The number of leaves at flowering of tomato WT **(A)**, *phyA* **(B)**, *phyB1* **(C)**, *phyB2* **(D)**, and *phyB1B2* **(E)** mutants grown under LD and SD conditions. Data are mean ±SE (*n* = 10 plants). **(F–J)** Expression levels of *SlSP3D, SlSP5G, SlSP5G2*, and *SlSP5G3* of tomato WT **(F)**, *phyA* **(G)**, *phyB1* **(H)**, *phyB2* **(I)**, and *phyB1B2* **(J)** mutants that were grown in LD or SD conditions. Data are expressed as means ±SE of three independent pools of extracts. Three technical replicates were performed for each extract.

As phytochromes are very important photoreceptors mediating flowering both in LD plants and SD plants (Izawa et al., 2002; Endo et al., 2005). We examined whether these photoreceptors have an effect on tomato flowering. We determined the number of leaves at flowering and the expression of the four expressed *FT*-like genes, *SlSP3D, SlSP5G, SlSP5G2*, and *SlSP5G3* in seedlings of *phyA, phyB1, phyB2*, and *phyB1B2* tomato mutants. We found that the number of leaves at flowering in *phyA* and *phyB2* mutants was the same as that in WT under both LD and SD conditions. However, the number of leaves at flowering in *phyB1* and *phyB1B2* mutants was 6 and 6.5, respectively, under LD conditions and this number was 8.5 on average under SD conditions (Figures 7A–E). In *phyB1* and *phyB1B2* mutants, there were a constant low expression level of *SlSP5G* under both LD and SD conditions (Figures 7H,J). However, there was a stable high expression level of *SlSP5G* in WT, *phyA* and *phyB2* mutants under LD conditions. Under SD conditions, there was a higher expression of *SlSP5G2* and *SlSP5G3* mRNA in *phyB1* and *phyB1B2* mutants compared to WT (Figures 7F,H,J). No difference was detected between *phyA, phyB2* mutants and WT on the expression levels of *SlSP5G, SlSP5G2*, and *SlSP5G3* under both LD and SD conditions (Figures 7F,G,I). Together, these results clearly demonstrate that PHYB1 has significant influence on the expression of *SlSP5G, SlSP5G2*, and *SlSP5G3*, and on the flowering time of tomato plants under both LD and SD conditions.

## Discussion

### *FT*-like genes act antagonistically to regulate floral initiation in tomato

Plant PEBP family proteins are divided into three major clades, with the FT-like and MFT-like clades primarily acting to promote and the TFL1-like clade primarily acting to repress floral development. In this study, we queried the complete tomato genome sequences and identified 13 PEBP genes, six of which belong to the FT-like clade, five are classified in the TFL-like clade and two are MFT-like clade. In the six FT-like clade, *SlSP3D, SlSP6A, SlSP5G, SlSP5G1, SlSP5G2*, and *SlSP5G3*, two, *SlSP6A* and *SlSP5G1* were not expressed in tomato plants. It has already been demonstrated that *SlSP3D/SFT*, the tomato ortholog of *FT*, induces flowering in day-neutral tomato and *sft* mutants show late flowering phenotype (Molinero-Rosales et al., 2004; Lifschitz et al., 2006). Here, we show that transgenic Arabidopsis plants possessing *SlSP3D* displayed much earlier flowering phenotype compared to control plants. FT-like proteins that promote flowering have been identified in many species such as *Populus* spp. (poplar; Böhlenius et al., 2006), *Malus domestica* (apple; Hättasch et al., 2008), *B. vulgaris* (sugar beet; Pin et al., 2010), *Solanum tuberosum* (potato; Navarro et al., 2011), *N. tabacum* (tobacco; Harig et al., 2012), and *Oryza sativa* (rice; Kojima et al., 2002).

Based on phylogenetic data *SlSP5G, SlSP5G2*, and *SlSP5G3* have been postulated to be orthologous to FT-like genes (Abelenda et al., 2014). However, overexpression of *SlSP5G, SlSP5G-2*, or *SlSP5G-3* in Arabidopsis resulted in late flowering phenotype compared control plants. In sugar beet and tobacco, FT-like genes can act as flowering promoters and repressors. The two sugar beet *FT*-like genes, *BvFT1* and *BvFT2* differ in three amino acid residues within the critical region encoded by the fourth exon (Supplementary Figure S1). Try (134), Gly (137), and Trp (138) are the most important three amino acids of the external loop for BvFT2 protein. Substitution of these three amino acid residues in BvFT2 was sufficient to convert it into a repressor (Pin et al., 2010). The change of BvFT1 Asn (138) into Try, Gln (141) into Gly and Gln (142) into Trp could completely revert its repressing function to promoting function in flowering. Four FT-like proteins have been reported in tobacco. NtFT4 is a flowering activator and the amino acid residues at the three conserved positions matched those of Arabidopsis FT and BvFT2 whereas the corresponding positions in the floral repressors NtFT1-3 were not conserved (Harig et al., 2012). Through protein sequence alignment, we also found that in the tomato SlSP3D the amino acid residues at the three critical positions were Tyr (133), Gly (136), and Trp (137) and these matched to those of the other floral activators, such as FT in Arabidopsis, BvFT2 in sugar beet and NtFT4 in tobacco (Supplementary Figure S1). However, the amino acid residues of SlSP5G in these three conserved positions were the same as those found in the floral repressors like NtFT1-3 in tobacco. The corresponding positions of SlSP5G2 and SlSP5G3 in these positions were not conserved compared with other floral activators and repressors (Supplementary Figure S1). These results suggest that SlSP5G, SlSP5G2, and SlSP5G3 were initially promoters of flowering but these mutations within the external loop converted its function to flowering repression. The three amino acids are critical for the activator vs. repressor function.

### The expression profiles of *FT*-like genes is influenced by photoperiod

The expression of *FT*-like genes in many species is regulated in a photoperiod-dependent manner (Samach et al., 2000; Kojima et al., 2002). Termination and flowering in cultivated tomato are not sensitive to day length, but flower initiation occurs earlier and inflorescence development far better in SD conditions than in LD conditions (Kinet, 1977). All four tomato *FT*-like genes were expressed exclusively in leaf tissue (Figure 2), which was the same as tobacco *FT*-like genes (Harig et al., 2012). In tobacco, *NtFT1, NtFT2*, and *NtFT4* showed higher expression levels under SD conditions than under LD conditions (Harig et al., 2012). In sugar beet, the floral repressor *BvFT1* was expressed at high levels when plants were grown in SD or in non-vernalized biennials plants that were not competent to flower (Pin et al., 2010). We also found that *SlSP5G* mRNA expression was up-regulated under LD conditions, while *SlSP5G2* and *SlSP5G3* mRNA increased under SD conditions (Figure 5B). The expression of *SlSP3D* was similar under both LD and SD conditions (Figure 5B). Although tomato is day-neutral with respect to flowering, the expression of the *SlSP5G, SlSP5G2*, and *SlSP5G3* identified here seem to be photoperiod dependent. *SlSP5G* most likely controls tomato flowering under LD conditions while *SlSP5G2* and *SlSP5G3* seem to regulate flowering under SD conditions. Tomato plants have an adaptive mechanism to adjust flowering according to photoperiod using a combination of different *FT*-like genes.

In this study, the silencing of *SlSP5G* by *TRV-VIGS* vector under LD conditions resulted in early flowering of tomato plants, and the silencing of *SlSP5G2* and *SlSP5G3* by *TRV-VIGS* vector under SD conditions led to early flowering of tomato plants. These results also showed that SlSP5G, SlSP5G2, and SlSP5G3 were floral repressors. SELF PRUNING (SP) is a homolog of TFL1-like gene and SP protein functions as an anti-terminator, maintaining vegetative growth (Pnueli et al., 1998). Mutant *sp* plants form progressively shorter sympodial units, until the shoots terminate in two successive inflorescences. In many species the ratio of floral activators and repressors, e.g., local ratios of SFT/SP3D (FT-like) and SP (TFL1-like), has been proposed to regulate local growth termination equilibria in all meristems of the tomato shoot system (Shalit et al., 2009; McGarry and Ayre, 2012). The three tomato FT-like floral repressors appear to have taken on the role usually played by TFL1 homologs in most other plants. Additional research is required to classify how FT-like floral activators and repressors and SP set the timing of the developmental switch from vegetative to reproductive growth. Both SFT/SP3D and SP of tomato bind to 14-3-3 and bZIP (SPGB, a homolog of FD) proteins in yeast, but each protein also has its own specific binding proteins (Pnueli et al., 2001). In Arabidopsis, FT protein is first transferred into the sieve elements and then subsequently transported by mass flow to the apex, where it interacts with FD to promote flowering (Abe et al., 2005; Wigge et al., 2005; Corbesier et al., 2007). In tomato, SlSP5G, SlSP5G2, and SlSP5G3 maybe like SlSP3D/SFT and they may interact with SPGB to control tomato flowering.

### Phytochrome B1 regulates FT-like genes

Phytochromes are photochromic proteins that regulate light responses under different light conditions (quantity, quality, and timing). Our data showed that, in the tomato *phyB1* mutant, the expression of *SlSP5G* under both LD and SD conditions was very low. The expression of *SlSP5G2* and *SlSP5G3* was always in a fairly high level, compared with WT under both LD and SD conditions. Based on the results we obtained in tomato plants, we found that the PHYB1 could promote the expression of *SlSP5G* under LD conditions but suppress the expression of *SlSP5G2* and *SlSP5G3* under both LD and SD conditions. It has been shown that PHYB has a general inhibitory effect on flowering in both LD plants and SD Plants (Lin, 2000; Yanovsky and Kay, 2003). An inhibitory effect of PHYB on FT expression has been shown in Arabidopsis (Valverde et al., 2004; Endo et al., 2005). In rice, the *phyB* mutation abolishes the night break effect on flowering and *Hd3a* mRNA, and PHYB suppresses the expression of *Hd3a* (Izawa et al., 2002; Ishikawa et al., 2005).

Phytochromes need to interact with the circadian clock to regulate flowering time in different day-lengths, but the molecular details of such interactions remains unclear (Valverde et al., 2004; Song et al., 2012). *phyB* mutations of the SD plant sorghum and the LD plant Arabidopsis both caused an early flowering phenotype; tomato *phyB*1 mutant also has an early flowering phenotype under LD conditions. One interpretation of this observation is that PHYB action may suppress floral initiation regardless of photoperiods, but the signal transduction or plant's responsiveness to PHYB signaling is gated by the action of the circadian clock, resulting in different day-length responses in the flowering time of different plants.

**Figure 8:**
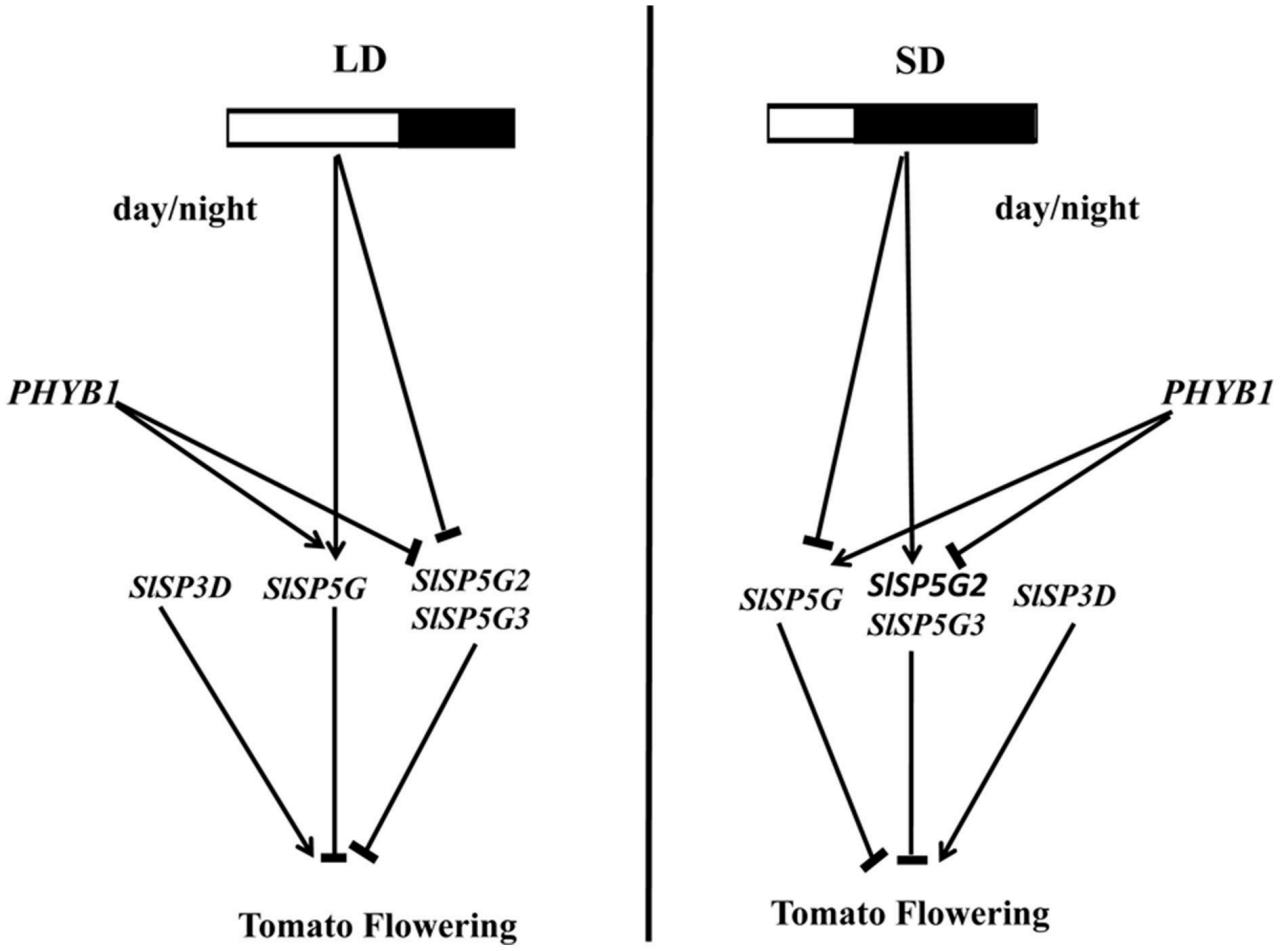
**Model of the photoperiod effect on flowering in tomato**. The expression of FT-like genes was regulated by photoperiod and mediated by phytochrome B1. In LD conditions, the expression of *SlSP5G* was induced, and the expression of *SlSP5G2* and *SlSP5G3* were inhibited. In SD conditions, the expression of *SlSP5G* was inhibited, and the expression of *SlSP5G2* and *SlSP5G3* were induced. The different expression pattern of tomato FT-like genes under different photoperiod may contribute tomato being a day neutral plant. Phytochrome B1 could promote the expression of *SlSP5G*, and inhibit the expression of *SlSP5G2* and *SlSP5G3*.

Based on the results we obtained in this study, we propose a model to explain the photoperiod effect on tomato flowering (Figure 8). This model is consistent with all of the results we obtained in our studies and suggests that the expression pattern of these *FT*-like genes is regulated by photoperiod and mediated by *PHYB1*. In addition, four tomato *FT*-like genes reveal they act antagonistically to regulate floral initiation. Understanding the molecular mechanism of flowering in a day-neutral plant has important implications for agriculture. Further studies are required to integrate the knowledge obtained from model species like LD plant Arabidopsis and SD plant rice to provide further insight on the mechanisms regulating flowering in day-neutral plant.

## Author contributions

Conceived and designed the experiments: SD and ZZ. Performed the experiments: KC. Analyzed the data: LC and KC. Contributed reagents/materials/analysis tools: XZ. Amplify the seed: LY. Wrote the paper: KC.

### Conflict of interest statement

The authors declare that the research was conducted in the absence of any commercial or financial relationships that could be construed as a potential conflict of interest.

## Supplementary material

Supplementary Table S1 **Sequences of primers used in this study for plasmid construction, quantitative RT-PCR and VIGS**.

Supplementary Figure S1 **Multiple amino acid alignment of PEBP domain of Arabidopsis, sugar beet, tobacco, and tomato PEBP family proteins**. The vertical arrowhead indicates the crucial amino acid change responsible for the difference between FT-like and TFL-like functions identified by Hanzawa et al. (2005). Amino acid residues conserved in FT-like proteins that promote flowering are shaded in red, identified by Pin et al. (2010) and Harig et al. (2012).

Supplementary Figure S2 **The exon-intron structures of tomato PEBP genes resembles that of AtFT**. Boxed areas depict the exons and lines represent introns. Numbers represent exon and intron lengths (bp).

Supplementary Figure S3 **The number of rosette leaves before flowering in the other *FT*-like genes overexpression lines**. All data are showed as mean ±SE of eight plants in each overexpression lines.

Supplementary Figure S4 **Silencing of PDS control gene causes photobleaching in tomato plans**. Photographs were taken 4 weeks after silencing. **(A)** Tomato plant infected by *TRV-SlPDS* vectors. **(B)** Tomato plant infected by empty *TRV* vectors.
