# Supplementary material for: Four Tomato FLOWERING LOCUS T-Like Proteins Act Antagonistically to Regulate Floral Initiation
Source: Front Plant Sci. 2016 Jan 11;6:1213. doi: 10.3389/fpls.2015.01213 (PMC4707262; doi:10.3389/fpls.2015.01213)
Supplement: Supplementary Table S1 — Sequences of primers used in this study for plasmid construction, quantitative RT-PCR and VIGS. [file Table1.DOC]

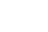


**Supplementary Table S1.** Sequences of primers used in this study for plasmid construction, quantitative RT-PCR and VIGS.

|  | |  |
| --- | --- | --- |
| Gene | | Primers (5’-3’) |
| For plasmid construction | | |
| SlSP3D | F | CATCGTCGACAATGCCTAGAGAACGTGATCCTC |
|  | R | AACTGCGGCCGCTCAATCAGCAGATCTTCTACGTC |
| SlSP5G | F | CATCGTCGACAATGCCTAGAGATCCTTTAATAGTTTCTG |
|  | R | AACTGCGGCCGCTTATAGGCGACGACCACCGG |
| SlSP5G2 | F | CATCGTCGACAATGCAAAGAGAAAGAGATACATTGAGAC |
|  | R | AACTGCGGCCGCTTATATTCTACGACCACCAGTACCA |
| SlSp5G3 | F | CATCGTCGACAATGAAGTTATATGTTATGAGTCCAAGGC |
|  | R | AACTGCGGCCGCTTATTCGCAACGACGACCAC |
| For quantitative RT-PCR | | |
| SlSP3D | F | GTTGGTCGTGTGGTAGGGGA |
|  | R | GCCTAAGCTCGCATCCATTA |
| SlSP6A | F | TTGATCCATTGATAGTTGGT |
|  | R | GCATTGTTCACTTCTCTATT |
| SlSP5G | F | CTAGCAACCCAAACCTGAGG |
|  | R | ATTGCCAAAGGTTGCTCCTG |
| SlSP5G1 | F | GCACTAATTTAAGGCCTTCT |
|  | R | GGATCAACCACAATCAGAGTATAA |
| SlSP5G2 | F | GATTTGAGGCCTTCTATGGT |
|  | R | ATCCACCATAACCAGAGTGT |
| SlSP5G3 | F | TTATATGTTATGAGTCCAAGGC |
|  | R | GTATTGAAATTTTGACGCCA |
| SlActin | F | ATTCCCTGACTGTTTGCTAGT |
|  | R | GCCATCTTATGCTATTCCTTTT |
| For VIGS analysis | | |
| SlPDS | F | CGCGGAATTCACTCAACTTTATAAACCCTGACGA |
|  | R | CATCGGATCCCAAACCATATATGTACATTTATCACAGG |
| SlSP5G | F | CGCGGAATTCTGGTCACAGATATCCCAGCA |
|  | R | CATCGGATCCTTTACATGTAGACTCGTATA |
| SlSP5G2 | F | CGCGGAATTCGATGCTCCAACTCCCAGTAATC |
|  | R | CATCGGATCCTCCGGGCTTTTAGAAAATATC |
| SlSP5G3 | F | CGCGGAATTCTTTCGTCAATTGGGAAGAGA |
|  | R | CATCGGATCC TTATTCGCAACGACGACCAC |
|  |  |  |
