# Supplementary figures and images for: Four Tomato FLOWERING LOCUS T-Like Proteins Act Antagonistically to Regulate Floral Initiation
Source: Front Plant Sci. 2016 Jan 11;6:1213. doi: 10.3389/fpls.2015.01213 (PMC4707262; doi:10.3389/fpls.2015.01213)

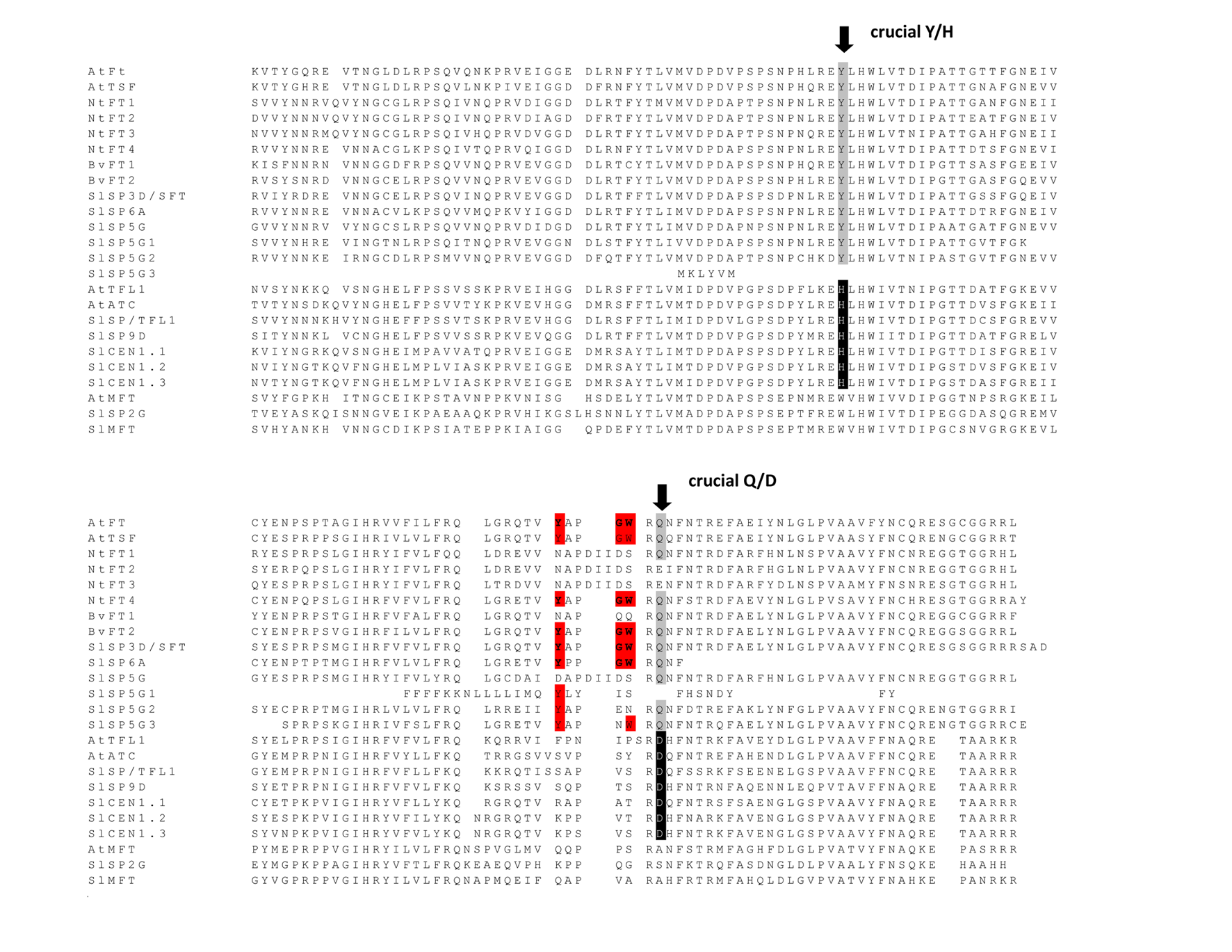

Supplement: Supplementary Figure S1 — Multiple amino acid alignment of PEBP domain of Arabidopsis, sugar beet, tobacco, and tomato PEBP family proteins. The vertical arrowhead indicates the crucial amino acid change responsible for the difference between FT-like and TFL-like functions identified by Hanzawa et al. (2005). Amino acid residues conserved in FT-like proteins that promote flowering are shaded in red, identified by Pin et al. (2010) and Harig et al. (2012). [file Image1.TIF]

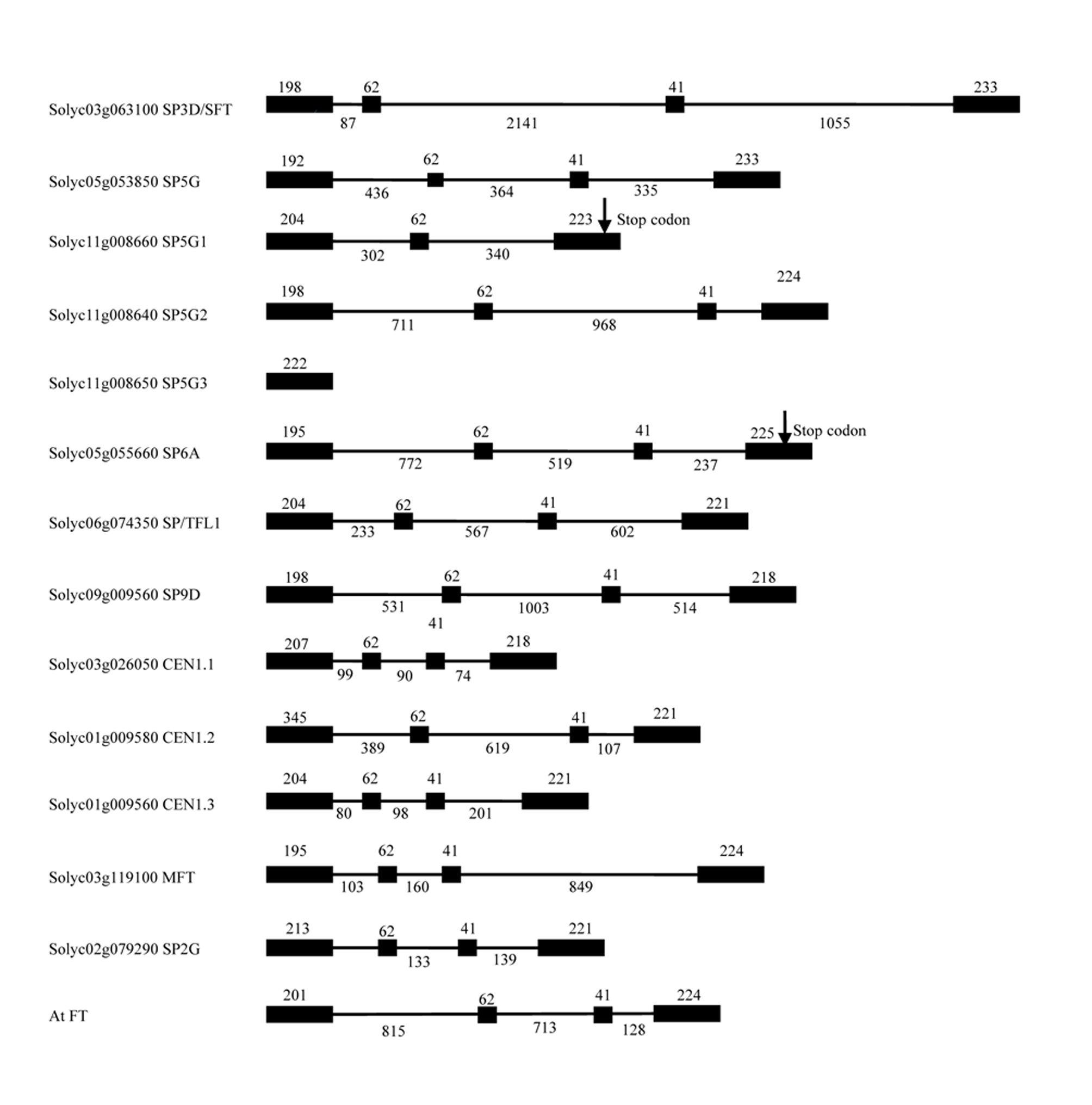

Supplement: Supplementary Figure S2 — The exon-intron structures of tomato PEBP genes resembles that of AtFT. Boxed areas depict the exons and lines represent introns. Numbers represent exon and intron lengths (bp). [file Image2.TIF]

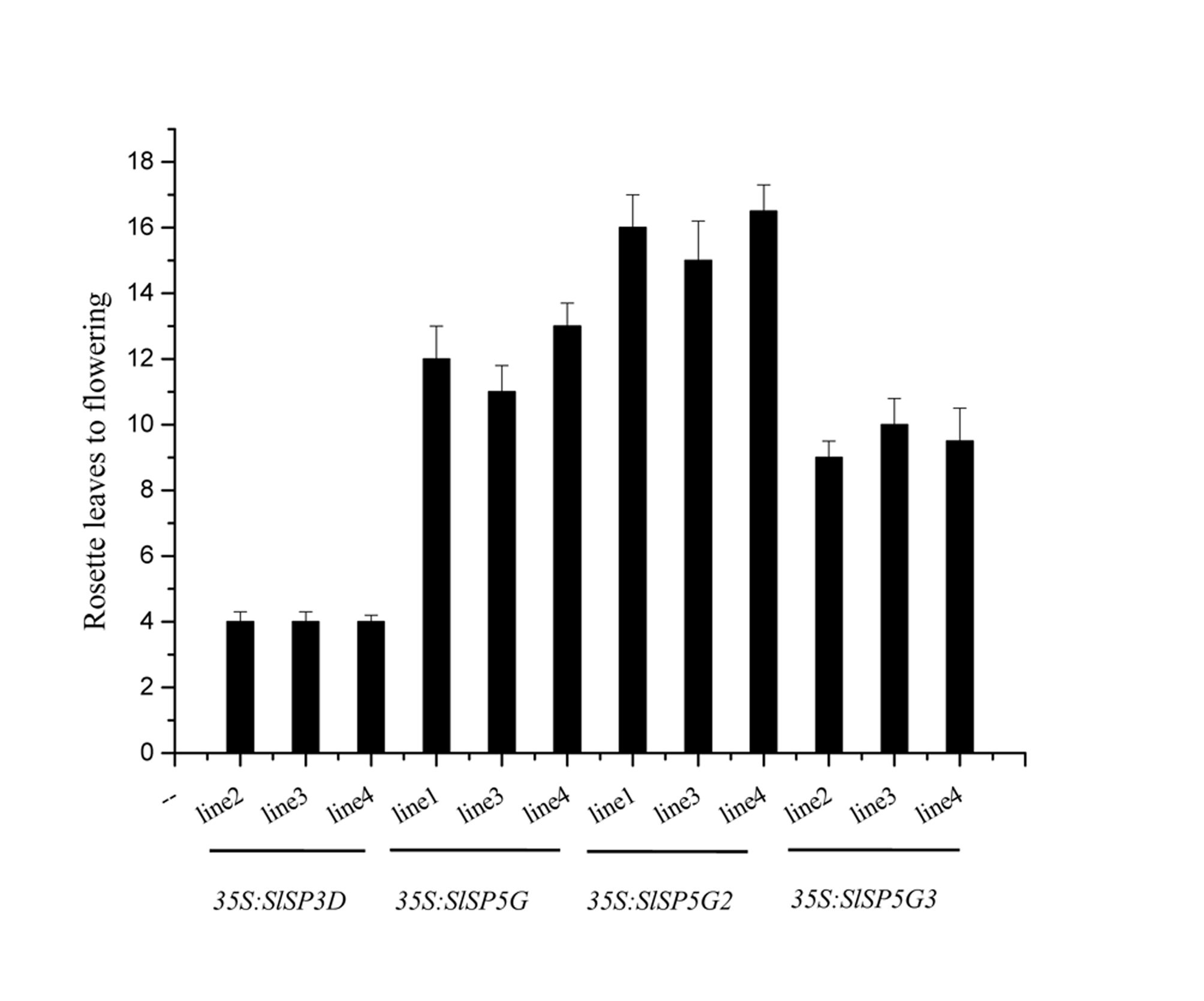

Supplement: Supplementary Figure S3 — The number of rosette leaves before flowering in the other FT-like genes overexpression lines. All data are showed as mean ±SE of eight plants in each overexpression lines. [file Image3.TIF]

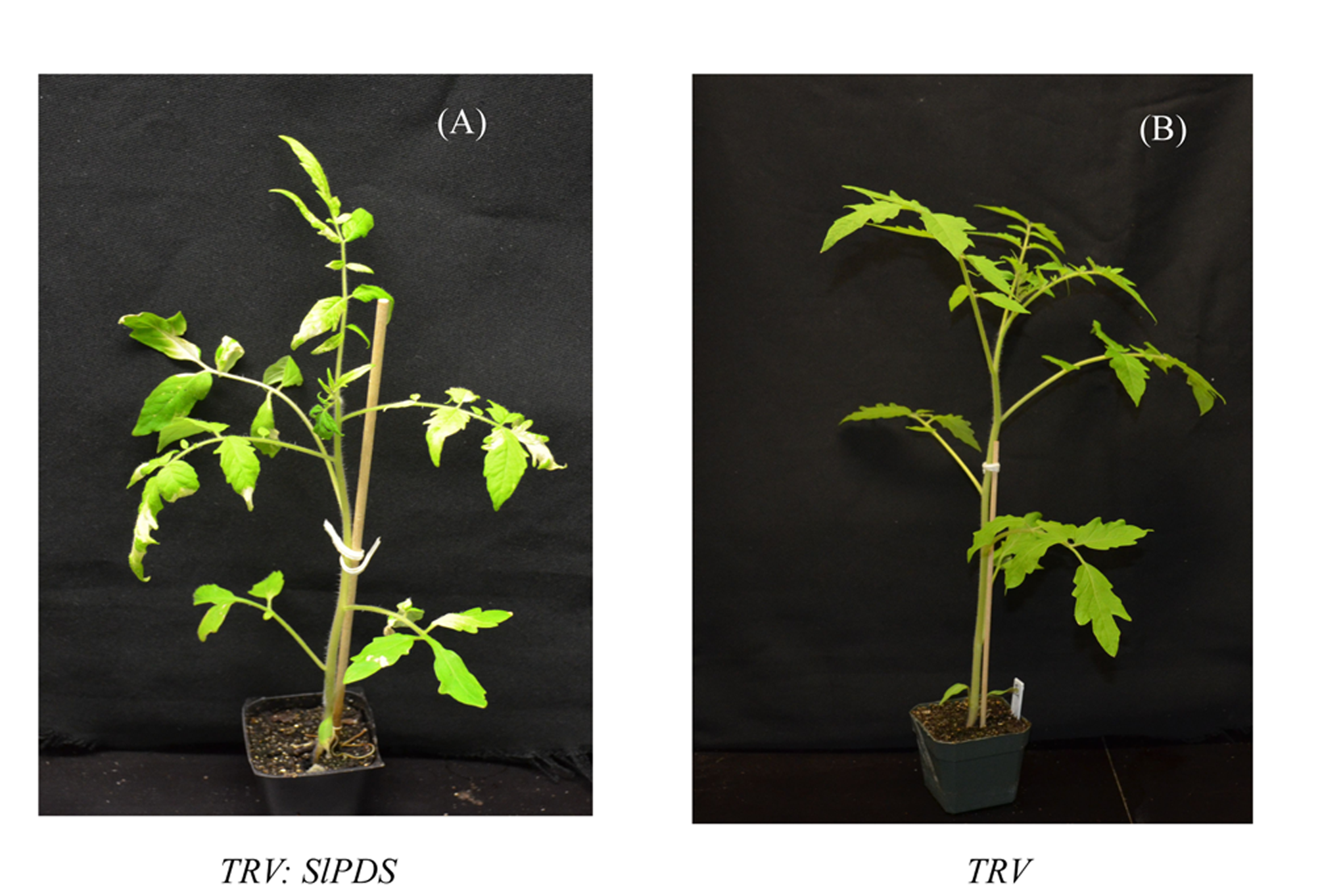

Supplement: Supplementary Figure S4 — Silencing of PDS control gene causes photobleaching in tomato plans. Photographs were taken 4 weeks after silencing. (A) Tomato plant infected by TRV-SlPDS vectors. (B) Tomato plant infected by empty TRV vectors. [file Image4.TIF]
